# Adherence to eHealth-Delivered Exercise in Adults with no Specific Health Conditions: A Scoping Review on a Conceptual Challenge

**DOI:** 10.3390/ijerph191610214

**Published:** 2022-08-17

**Authors:** Andrea Fuente-Vidal, Myriam Guerra-Balic, Oriol Roda-Noguera, Javier Jerez-Roig, Joel Montane

**Affiliations:** 1 Research Group on Health, Physical Activity and Sport (SAFE), Blanquerna School of Psychology, Education and Sport Sciences, Universitat Ramon Llull, 08022 Barcelona, Spain; 2 Research Group on Methodology, Methods, Models and Outcomes of Health and Social Sciences (M_3_O), Faculty of Health Sciences and Welfare, Centre for Health and Social Care Research (CESS), University of Vic—Central University of Catalonia (UVic-UCC), 08500 Vic, Spain; 3 Fundació Naccari-Ravà, 08036 Barcelona, Spain; 4 Blanquerna School of Health Science, Universitat Ramon Llull, 08025 Barcelona, Spain

**Keywords:** eHealth, mHealth, smartphone, physical activity, exercise, adherence, engagement, attrition, apps, digital health, treatment adherence and compliance

## Abstract

Adherence has emerged as a focal point and critical determinant of success for physical activity interventions. The term is used for both traditional and digital interventions, and for prescribed and nonprescribed activities. Many other terms for adherence are being used interchangeably, as there is no consensus on its precise conceptualization. This scoping review aimed to advance the definition of adherence to eHealth programs, specifically for the adult population with no specific health conditions. A total of 2983 papers, published between 1 January 2016 and 13 March 2022, were retrieved from different databases (including grey literature). Of those, 13 studies met the eligibility criteria and were included for review. The selected studies used a wide array of technologies and consisted mainly of exercise interventions. Most of the reviewed publications contemplated exercise adherence as a percentage of expected dose. Most (8 out of 13) studies neither assessed nor specified an expected use of the involved technology. Results suggest a need for homogeneity in the conceptualization of adherence to physical activity and exercise, including those interventions delivered digitally.

## 1. Introduction

The beneficial effects of active living and regular physical exercise have a solid evidence base [1]. The World Health Organization (WHO) has consequently made it a worldwide public health priority to increase physical activity and to concurrently decrease sedentary behavior [2]. In spite of this, Guthold et al. established a global age-standardized prevalence of insufficient physical activity of 27.5% in 2016, with a difference between sexes of more than 8 percentage points (23.4%, 21.1–30.7, in men vs. 31.7%, 28.6–39.0, in women) [1].

Research and innovation funding programs such as Horizon Europe have established tools, technologies and digital solutions for health and care as their main areas of intervention [3]. In an ever more digitalized world, robust monitoring and evaluation plans for digital interventions have been specifically identified as essential to support potential intervention scale-up [4]. In both digital and nondigital physical activity research, adherence has been emerging as a focal point and relevant variable [5,6,7,8]. Adherence to eHealth technology is an underdeveloped and often improperly used concept in the existing body of literature [7]. One of the most persistent issues in the consistent operationalization of the term adherence is the lack of a clear definition of this concept [5,6,7,8], especially when a medical prescription or otherwise preset exercise calendar is not involved. However, even for the most structured therapeutic exercise interventions, an agreed communicable definition of adherence is lacking [5]. The latter constitutes a problem, since it prevents patients and healthcare providers from working toward a shared goal while hindering measurement and the ability to monitor its variability [5]. All of these barriers become considerably more relevant when the term adherence is applied to voluntary (nonprescribed) activity, when recommendations or assessments lack specificity (e.g., exercise volume), and when technology is involved—which often leads to a duplication of adherences (i.e., adherence to the expected behavior vs. adherence to the technology usage). In terms of comparability between studies, an additional barrier is the fact that compliance is often defined as a binary variable, using a combination of two outcomes (e.g., 10 h a day for 3 out of 7 days) [9].

In summary, as long as a valid, reliable and acceptable measure of adherence to different physical activity or exercise interventions is not agreed upon, interventions or methods for improving adherence may be questioned [5].

### 1.1. Adherence to Physical Activity (PA)

Research has commonly focused on the assessment of adherence to PA, as per the physical activity guidelines by the WHO, both for people with and without pathological conditions. The WHO’s 2020 PA guidelines recommend that adults undertake at least 150 min of moderate-intensity (or an equivalent combination) of physical activity per week, plus 2 or more days of muscle strengthening activities for additional health benefits [10]. When using the WHO—or any other recognized institution—as a reference, adherence can be easily categorized as a binary variable (i.e., user is either adherent or not adherent to the recommended levels of PA).

In those cases where the study design specified particular PA goals (different to those in guidelines), authors commonly categorized adherence as good when objective behavior equaled 80% or more of the planned behavior [5]. Several studies were in line with this approach [11], most of which involved individuals with health conditions. When PA was programmed, attainment (as opposed to adherence) was generally high. According to Sperandei et al., this could have been keeping the proportion of active people stable while at the same time failing to ensure individuals participate long enough to achieve their objectives [12].

### 1.2. Adherence to Exercise

Whereas PA includes all skeletal body movements and energy expenditure, it can only be categorized as exercise when it is “planned, structured, repetitive and purposefully focused on improvement or maintenance of one or more components of physical fitness” [13].

Adherence to exercise is a widely used term, subject to a range of interpretations. It has been estimated that 60% of research studies on therapeutic exercise do not provide a clearly identifiable definition of adherence [5]. Additionally, even when they do, it is often constructed ad hoc [5], a fact that has led authors to recommend that a more consistent and standard validated measure of exercise adherence be developed [14].

A systematic review, by Bailey et al., on exercise adherence for musculoskeletal pain disorders pointed out that most of the reviewed studies defined adherence as a function of the parameters used for its assessment, with frequency being the most commonly measured parameter [5]. In this regard, frequency can be interpreted as a predetermined cut-off or as a distribution method, which explained why adherence levels ranged from 15% to 100% completion of the prescribed exercises [5]. While frequency is certainly a useful parameter, the authors highlighted that other parameters, such as intensity, time, accuracy or behavioral components, could also contribute to adherence and would be worth considering [5].

In clinical settings, adequate adherence to physician instructions has generally been reported to be low, with values ranging from 30% to 60%, depending on the complexity of the behavior changes required [15,16]. Most research on exercise has been conducted while focusing on structured exercise programs, often supervised and commonly performed within clinical settings [14]. When it comes to the temporal dynamics of adherence in unsupervised/unstructured settings, there is an evident gap in the literature [12].

In the context of fitness centers, for instance, the probability of an individual maintaining their membership uninterrupted for more than 12 months has been estimated at around 5% [12].

### 1.3. Adherence to eHealth

A 2021 systematic review on digital health usage associations concluded that a small but significant positive relationship existed between engagement with a digital health intervention and PA outcomes in healthy adults [17]. The WHO itself recommended that digital health interventions (DHI) be used to promote and support participation in PA [2]. Accordingly, an increase of 26% in article publications on DHI to improve PA was documented between 2000 and 2018 [18].

In contrast, evidence up until 2017 highlighted the fact that many eHealth evaluations lacked or did not report positive effects [7]. The same systematic review established that the interventions were often not used or were abandoned after a period, which, in either case, resulted in the available technological elements not being used as intended by their developers [7]. Within a broader spectrum—and considering digital behavior change interventions as a whole usage—levels have been found to be typically low [4,19]. Informal data suggest that 26% of apps are used only once after downloading and only 26% of users access the app more than ten times [20]. Our own results, from the evaluation of a popular commercial fitness app, showed that only 17% of subscribers actually completed their first workout and as few as 2% of initial enrollments reached practical session number eight [21].

When it comes to electronic interventions, the traditional definition of adherence—based on completion of the prescribed intervention—seems to fall short. Several research papers, including a series of systematic reviews, have drawn attention to the heterogeneity of definitions and the need to unify criteria [6,7,8]. Variables most often used to measure adherence to eHealth interventions include number of logins, number of days of technology use, time spent on the platform, number of lessons or modules accessed or finished, and number of elements accessed or used [7,17].

To our knowledge, three authors, in particular, have attempted to categorize adherence to health interventions delivered through electronic means. In 2017, Sieverink et al., determined that there were three requisites for measuring adherence to eHealth interventions. Those included: 1) that the design provides a justification (empirical, theoretical or rational) of the intended use; 2) that operationalization of intended use is provided; and 3) the ability to measure actual usage [7]. Consequently, the authors operationalized the definition of adherence in three different categories: Category A was assigned when an intended use of a technology was not specified and adherence was operationalized in terms of “the more usage, the better”. Category B was assigned when intended use of a technology was specified but not justified (for example: “a user is adherent when logging in at least once a week for three subsequent weeks”). Category C was assigned when the intended use of the technology was provided, together with some form or rationale [7]. A year later, Payne et al. conducted a narrative review on adherence to dietary self-monitoring using a mobile app [6]. Their results indicated that adherence was operationally defined in two ways: as either adherent or nonadherent, or by frequency (of dietary intake recording, interaction with apps, timing of recording), with some studies simultaneously using both definitions [6]. Finally, data published by Yang et al., in 2020 provided similar insights in regard to PA app adherence [8]. Further detail is provided in the Adherence to eHealth for General PA and Exercise, below.

### 1.4. Adherence to eHealth for General PA and Exercise

Increased engagement in DHI has been suggested to have a weak but positive relationship with PA outcomes, in terms of both objective and subjective experiences in adults [17]. Augmenting PA levels in all populations has been set as a priority [2] and eHealth has the potential to address this matter, potentially with high effectiveness and at a low cost [22]. A recent systematic review revealed that interventions using eHealth could strongly increase PA levels and reduce sedentary time among inactive participants [23]. Among the many appealing features of DHI, an important factor is their capacity to be scaled for larger populations [8,24].

It is widely accepted that motivation is a determinant of behavior change. It has been pointed out that continuous use of eHealth could make this feasible and cost-effective [23]. In a fitness facility in Brazil, weight loss was found to be the most prevalent motivation to start an exercise program [12]. However, it was also found to be significantly related to higher chances of an early drop out [12]. Exploring baseline motivation has been common practice, but Sperandei’s results indicated that the potential changes in motivation during intervention could be highly significant, and that looking at motivation alone does not seem sufficient to promote adherence [12]. As far as technology is concerned, we now know that app glitches are able to profoundly influence app adherence in a negative manner [8], yet researchers seem to be lacking a consensus of conceptualization for adherence [7,8,12,25] and other related terms.

Research participants have traditionally self-reported their activity by using logs [5]. More recently, fitness trackers, pedometers and accelerometers have become popular instruments for obtaining reliable estimates of PA [26]. Some of these technologies, alone or in combination with others, allow researchers to measure not only wear duration and step count, but also other relevant factors related to exercise, such as activity intensity and energy expenditure [25]. Even for activity tracking publications, heterogeneity in the analyses and reporting of data has been observed—and authors have recommended that minimum reporting thresholds be determined in the future [25].

It has been suggested that whenever PA/exercise practice is linked to the use of eHealth, it is not sufficient to promote the effects of the technologies without an accompanying assessment of its influence on the intervention [27]. This should be borne in mind, as it has been shown that more complex behavioral recommendations tend to lead to higher nonadherence rates [16]. In an attempt to tackle this, researchers focused on assessing adherence to the prompts delivered via health technologies and found that it tended to diminish over time [28].

In a 2020 scoping literature review on PA app adherence, Yang et al. were able to establish a total of four possible category dimensions for adherence to PA apps: (1) frequency of PA app usage, (2) intention/motivation to sustain use of the PA app, (3) degree of function use within the PA app, and (4) the duration of PA app usage [8].

The explanations above on the particularities of eHealth intervention design point to the existing split between traditional adherence to exercise and adherence to eHealth-delivered exercise, where the technology itself can be a determining factor in intervention effects. A standard, validated measure of exercise adherence that can be consistently used in future studies is required [14]. This review aimed to offer insight into how adherence to eHealth-delivered exercise has been utilized in recent scientific literature. Being aware of past conceptualizations could constitute a first, necessary step toward increased homogeneity in research—and could allow clinicians and researchers to analyze and report in a more standardized and replicable manner.

## 2. The Goal of the Review

The primary goal of this scoping review was to analyze and report how researchers have approached the definition and operationalization of adherence to physical activity interventions via eHealth for the adult population with no specific health conditions. This subsample of the population constitutes a particularly challenging group, since their access to interventions is often voluntary, lacks the urge of a health-specific need and, most importantly, lacks a formal medical prescription. These characteristics, together with the fact that the interventions were delivered via technology, meant that these scenarios do not fit as easily into the traditional definition of adherence—the percentage of actual compliance over the prescribed dose.

The broad directive in this review was to determine whether the inclusion of e-Health technologies in physical activity/exercise interventions required a new conceptualization of the term adherence. The research question was: how has eHealth exercise adherence for the healthy adult population been defined and operationalized in the literature over the last six years? These questions directed the subsequent search and selection of relevant e-Health publications presented in this review. This paper include the review findings and accompanying analysis, which together provide insight into how adherence to exercise has been operationalized in the past, both for in-person and electronic exercise plans.

## 3. Methods

This scoping review followed the stages as outlined by Levac et al. [29] in their update to the work of Arksey and O’Malley in 2005 [27]. Six steps to the review process were followed: (1) identifying the research question, (2) identifying the relevant studies, (3) study selection, (4) charting the data, (5) collating, summarizing and reporting results, and (6) optional consultation [29]. This framework recommended that scoping reviews employ both a broad and a more focused questions for step one [29].

### 3.1. Protocol and Registration

This review was not previously registered, as it is not customary to do so with scoping reviews. PROSPERO, an open access online database of systematic review protocols and registry for systematic review protocols, was nevertheless consulted (with the search terms “adherence”, “physical activity”, and “exercise”) in order to determine whether any similar reviews were ongoing. Sixty-one related publications were found, of which eleven were initially perceived as relevant to our study. After a thorough reading of their designs, it was determined that none of them matched or were otherwise relevant to our study. Five were disregarded for not involving technologies and the remaining six were found not relevant for involving patients with pathologies.

### 3.2. Search Strategy

**Figure 1:**
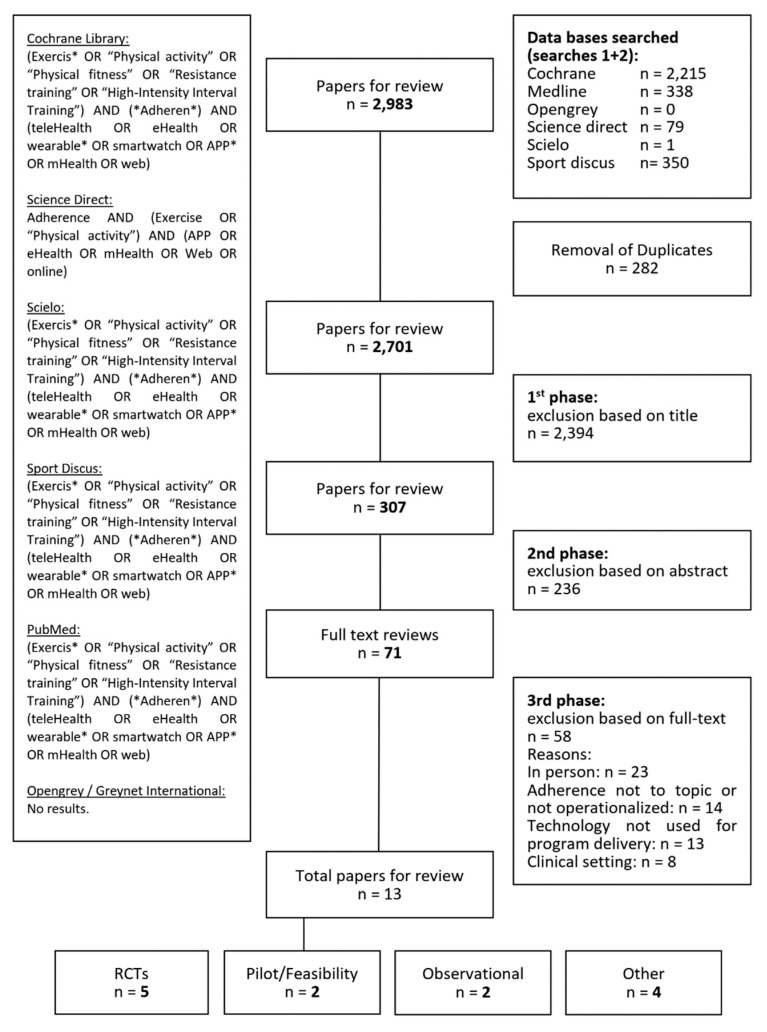
Scoping review strategy and results.

A preliminary search strategy was used during step two to determine the medical subject headings (MeSH) and keywords. The portal-related search terms were separated into four categories: “telemedicine”, “exercise”, “physical fitness”, and “treatment adherence and compliance”. A combination of the constructs and related keywords was used for a comprehensive literature search using the Cochrane Library, Science Direct, Scielo, PubMed, Sport Discus, and Greynet International databases. The exact search terms used can be found in Figure 1. An additional manual search was performed to complete the review, and spontaneous findings were not systematically disregarded. Papers were searched regardless of their study design, language, and publication status.

The literature search was conducted at two different points in time, using the same search queries on both occasions. The first search was carried out during February 2021 and encompassed published articles from 1 January 2016 to 31 December 2020. The second search was carried out in March 2022 and was restricted to publications from 1 January 2021 until 13 March 2022, in order to better reflect the impact of the COVID-19 pandemic.

A detailed description of keywords and search queries for each database is provided in the Multimedia Appendix A.

### 3.3. Eligibility Criteria

In the third step of the review process, inclusion and exclusion criteria were used. All articles that met or seemed to meet the following criteria were included in the review: (1) involved humans, with no specific health conditions; (2) subjects were 18 years of age or above; (3) intervention consisted of physical activity or exercise, delivered or supported remotely; (4) the article specifically mentioned adherence, referring to exercise, physical activity, sedentary behavior, or app use. Articles which did not fulfill the inclusion criteria were excluded, as well as those in the following situations: (1) if the article dealt with pathology or related problems (e.g., referring to pathology, therapeutic, rehabilitation, chronic conditions, painful conditions, disability, frail, fragility, clinical, hypertension, prescription, critical, disorders, primary care, doctor, nurse, practitioner); (2) adherence was not conceptualized, defined, or measured; (3) adherence referred to variables other than physical activity, sedentary behavior, exercise, or app use (e.g., diet, combined interventions, activity tracker wear time); (4) studies relating to pregnancy or performance sports.

### 3.4. Data Extraction

The studies (*n* = 13) selected for review were screened for data, and key points were extracted and registered in a Microsoft Excel sheet (MS Office 16). This constituted step four (charting the data) of the scoping review framework process. Characteristics such as author, title, country of origin, research design, year, journal of publication, and outcome measured were recorded.

## 4. Results

This section reflects the concluding steps of the framework: collating, summarizing, reporting results and consultation. Next, the discussion section was utilized to expand on some areas.

### 4.1. Study Selection and Description

The technology-based search rendered a total of 2983 (2616 from the first search and 367 from the second search) relevant studies. Selection of studies was manually conducted, on an independent peer-reviewed basis, by the authors (AFV and JMM). Duplicates (282 total, 263 in first search and 19 in second search) were initially identified and excluded from the list. The selection of studies was completed in three steps and carried out simultaneously by both authors. First, titles were screened, and most (2394) papers were excluded for having to do with pathological/clinical scenarios. Second, the abstracts of the 307 articles deemed relevant were screened for eligibility, also on a peer-reviewed basis. Two hundred and thirty six papers were discarded based on the information on their abstract. Lastly, those papers that could not be excluded upon abstract check (58 from first search and 13 from second search) were deemed eligible for full text reading. In total, 71 papers were fully read and 58 of them were excluded during the full-text screening phase (Figure 1). All-stage decisions by the authors as to whether papers should be included or excluded for review were placed in common at the end of each stage. Disagreements were discussed one by one until consensus was reached. A total of 13 studies (6 studies from the first search and 7 studies from the second search) studies were finally deemed eligible. Most full texts (*n* = 23) were excluded due to their interventions being delivered in person and not by means of eHealth technologies. Only papers which included the use of technology for either the implementation of a PA/exercise intervention or its follow-up were considered eligible. A list of all reasons for exclusion is presented in Figure 1 for review.

The selected studies included 5 randomized controlled trials (RCTs), 2 pilot or feasibility studies, 2 RCT protocols, 2 observational studies, 1 formative research, and 1 congress abstract. Table 1 summarizes the key characteristics in the selected studies. Issuing countries included Australia [30,31], Greece [32], Spain [33,34], The Netherlands [35], Germany [36], United States of America [37,38], United Kingdom [28,39], China [40] and Taiwan [41]. A noticeable increase in the number of publications could be seen for 2021 and 2022.

### 4.2. Range of Conceptualizations for Adherence in the Reviewed Papers

**Figure 2:**
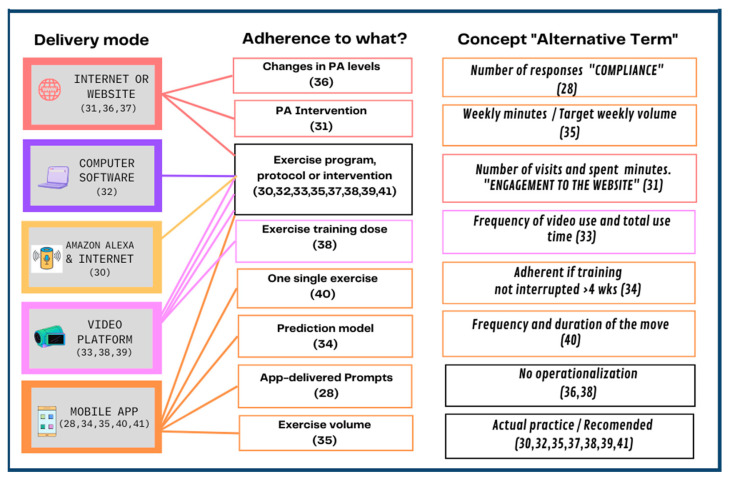
How has technology-delivered physical activity/exercise been defined in the literature [28,30,31,32,33,34,35,36,37,38,39,40,41].

In general, this review comprised a range of technologies and measures applied to the evaluation of adherence (Table 1). The technologies used included computers [32], internet or websites [31,36,37], Amazon Alexa and internet [30], video platforms [33,38,39], and mobile applications [28,34,35,40,41]. See Figure 2.

As is customary in scoping reviews, the nature of the studies reviewed was heterogeneous, not only due to the different technologies used but also in regard to the choice of intervention, delivery, and registry mode, among other things. Most (8 out of 13) of the reviewed articles assessed adherence to exercise programs, protocols or interventions [30,32,33,35,37,38,39,41]; one focused on adherence to changes in PA levels [36], one focused on adherence to app-delivered prompts [28], one assessed adherence to a single exercise [40], one measured adherence to a PA intervention [31], and one focused on establishing a prediction of adherence based on a previously published pilot study [34]. In addition to measuring adherence to the exercise program, one of the studies also aimed to assess adherence to exercise volume [35] and yet another aimed to assess adherence to the exercise training dose [38]. Other than that, still related to the concept of adherence, one of the reviewed papers additionally measured engagement on a website, as the number of visits and minutes spent on it [31].

The majority of the reviewed papers that focused on assessing adherence to exercise programs did so by operationalizing the term as a percentage of actual practice over the recommended or planned amount of practice [30,32,35,37,38,39,41]. Of those, one of the studies specified that calculations had only been done for those participants who had completed the intervention [37]. A similar operationalization (i.e., percentage of actual versus planned participation) was used to assess adherence to PA by Alley et al. [31]. Only one of the papers which focused on adherence to an exercise program conceptualized the term adherence based on the mere frequency of video use and total use time [33].

One of the studies included in this scoping review did not effectively specify their operationalization of adherence [36], although they did mention that they focused on assessing changes in PA levels, while holding the WHO guidelines as the standard recommendation. One study focused on exercise volume adherence, by operationalizing it as the number of weekly minutes the person exercised, divided by the target weekly volume [35]. Another study reported adherence to exercise training dose and specified it would be monitored through heart rate-based training zones [38]. However, the precise operationalization of adherence in this case remained unclear.

One study operationalized adherence to a suggested single exercise by the frequency and duration of the move (handgrip) [40]. The study by Morris et al. focused on adherence to app-delivered prompts, as opposed to app-delivered exercises. They interchangeably use the term “participant compliance” and operationalized adherence to the prompts as the total number of participant responses [28]. Lastly, the study by Jossa-Bastidas et al. consisted of a deep-learning-developed prediction model for adherence to a given exercise app. The authors, in this case, operationalized adherence as a binary variable, according to whether the participant interrupted their training for a period of 4 weeks or more (nonadherent) or not (adherent) [34].

### 4.3. Adherence Operationally Defined as Related to Technology Design

Table 2 provides details as to how each of the reviewed articles categorized adherence to the technology, based on recommendations by Yang et al. [8] and Sieverink et al. [7]. Detailed information on these categories has also been included in the Adherence to eHealth for General PA and Exercise section, above.

The authors were unable to categorize any of the reviewed articles into any of the four categories described by Yang et al. in 2020 [8], as reflected by the n/a (not applicable) indications in Table 2. In most (8 out of the 13 reviewed studies) cases, the reason for this was that the reviewed articles evaluated interventions delivered by technology types other than mobile applications (i.e., computers, internet, Amazon Alexa, or videos), which, in essence, were not within the aim or scope of Yang’s [8] publication. Other reasons included, in two instances, the app not delivering the intervention, in two instances, adherence to the app not being assessed or reported, and in one instance, the study not actually being of an interventional nature.

In regard to the classification of findings according to the eHealth categories established by Sieverink et al. in their 2017 systematic review [7]: most (8 out of 13) studies fell within Category A, meaning that an expected use of the technology was not specified in the reviewed papers so that a ‘the more use, the better’ approach was assumed. Of those articles which fell into Category A, one of the interventions was computer-delivered [32], two were internet-based [31,36], two were app-based [28,41] and three consisted of videos [33,38,39]. One paper, which involved an internet-delivered intervention [37], was identified as falling within Category B. One paper [30] offered explanation of intended use and justification for that choice, therefore falling into Category C. Three papers [34,35,40] could not be classified into any of Sieverink et al.’s pre-established categories.

## 5. Discussion

The purpose of this scoping review was to gain insight into how the concept of adherence has been used in previous eHealth interventions involving healthy adults. Our initial hypothesis, based on sound previous research, was that the term adherence was used in a variety of forms, conceptualizations and or definitions, therefore hindering direct comparison of measurements between studies. We began this research with the question of whether the trend toward eHealth interventions required an update of the traditional definition of the term adherence. Based on our observations and also on those of other researchers [6,7,8], both assumptions (i.e., variety of uses of the term adherence and a need for reconceptualization in light of increased technology use in healthcare) seem to have been confirmed.

### 5.1. Principal Findings

We included 13 studies in this review, all published between 1 January 2016 and 13 March 2022. Most interventions were delivered via smartphone application, followed by video platforms. A drift toward apps seemed apparent as we compared our results with those of Sieverink et al., who found that up until 2017, most digital interventions were web-based or mobile apps. Eight out of the thirteen studies reviewed focused on adherence to exercise programs. The fact that they most commonly included some kind of suggested program pinpointed a gap in the literature as far as voluntary, nonprescribed exercise was concerned. Our results also highlighted the fact that operationalizations of the term adherence were often based on the assumption that more use was better and did not include a threshold for intended use. This seemed to be in line with Sieverink et al.’s findings from 2017. We were surprised to find that few of the studies we reviewed were specific on the expected use of the technology, in contrast to the results previously found by Sieverink et al., in their review.

Most of the studies included in this scoping review measured adherence as the percentage of actual practice over the recommended or planned amount. Indeed, the most extended conceptualization of adherence in the published literature was that of the WHO, which established it as “the extent to which a person’s behavior—taking medication, following a diet, and/or executing lifestyle changes—corresponds with agreed recommendations from a health care provider” [42]. This definition could be somewhat confusing, since adherence to prescribed therapies or recommendations is also known as compliance, as per the original conceptualization by Sacket et al. [43,44], which then led to the WHO implementing a new iteration of their definition. The WHO’s preference for the term adherence aimed to better reflect the autonomy of the patient and the influences of their environment, experiences, knowledge, or resources [5]. Within our own results, the term compliance was found to be used interchangeably with adherence on one occasion.

Previous evidence has pointed at many other confusing terms being commonly used in research. In the past, exercise adherence has been used interchangeably with the terms intervention adherence, study adherence [45], commitment [46], retention [30,47,48], compliance [44], consistency [49], engagement [17,50,51], and feasibility [52]. When studying adherence to therapeutic exercise for musculoskeletal pain, Bailey et al. additionally found other terms to have been used interchangeably, such as concordance, agreement, cooperation, partnership, and therapeutic alliance [6]. The scientific community also lacks a clear operationalization of the term adherence to physical activities, even in clinical settings. Most studies which have defined adherence in their work have used frequency of exercise completion as the measuring parameter of adherence [5]. However, even frequency was not always measured the same way; it consisted variably of exercise repetitions, blocks of exercise time, or a variety of time frames (e.g., exercises per day, week or month) [5]. Other studies in the field of musculoskeletal pain have measured adherence based on behavioral parameters, session attendance, session completion, exercise exertion, or intensity or quality of execution [5]. Moreover, there is a third term that should be brought to mind; if adherence is the amount of exposure that people receive when using an intervention, then it could also be referred to as dose [46]. To this regard, Cugelman et al. considered that, for interventions that were voluntary, users received a dose that was proportional to their chosen level of adherence [46]. This is just another example of the terminological jigsaw puzzle researchers face, which will hopefully be solved with more standardized scientific reporting. Moreover, none of the included studies dealt directly with all four parameters (i.e., frequency, duration, intensity and accuracy) that the WHO believed characterized rehabilitation prescriptions [5].

This paper showed that the reviewed interventions frequently lacked a justification of the exercise prescription. This was also the case in Sieverink’s review [7], which spanned from 2006 to 2017. In some cases, the reviewed papers did provide some type of intervention rationale. Albergoni [35], as well as Konstantinidis [32], specified that they were adhering to published PA guidelines. Sun [41] explained that they devised their own exercise plans. Even then, when talking about eHealth, researchers encountered yet another challenge: being able to differentiate between adherence to the intervention and adherence to the technology being used. In fact, 8 out of the 13 studies reviewed did not specify any intended dose of use of the technology.

### 5.2. Implications and Recommendations

This scoping review identified ongoing challenges regarding the conceptualization of the term adherence in health, and eHealth in particular. Exercise implies a personalized objective setting, which adds to the difficulty of reaching some homogenization of the concept. We seem to be facing a need to develop specific definitions for specific scenarios (according to purpose, population group, voluntary vs. prescribed, etc.).

Technology plays an important role and could be valuable in obtaining objective usage data. However, for eHealth interventions to be successful, future researchers and developers should focus on user perception of technology use—for instance, by using the System Usability Scale or a similar tool.

While some consensus for the use of the term adherence was reached, the authors of this review recommend that future studies clearly specify characteristics of their chosen technology, operationalization, expected user behavior toward both the intervention and the technology, and the existence or absence of a rationale to their selections.

## 6. Strengths and Limitations

In the conduction of the scoping review, the authors faced a number of limitations worth taking into consideration. Regarding the search query used, we started with the realization that MeSH did not have an adherence term that focused specifically on life behavior change, which required more creativity and therefore a risk of bias. Additionally, the MeSH terms “exercise therapy” and “telemedicine” were avoided, given that their definition was circumscribed to pathological conditions. Excluding those papers focusing on health issues, pain, or other rehabilitation environments may have caused some selection bias. Similarly, the MeSH terms “sports” and “physical fitness” were also avoided. We made every effort to include as many papers and study designs as possible in our scoping review and decided, when in doubt, to include studies. However, we were compelled to slightly change the search equations for different databases, which, in itself, constituted some limitation to the procedure. Another limitation arose from the fact that we specifically searched for the term adherence, while other authors may have published work using different terms to refer to the same user behaviors. Additionally, the search terms were limited to title, abstract, and keywords for a number of databases. Restricting the search in this way enabled us to select those studies that specifically focused on adherence to PA/exercise in eHealth, but could have left some undetected.

This scoping review encompassed a timespan of 6 years and 3 months. The authors considered this adequate, as it constituted a follow-up to Sieverink et al. and their 2016 work. Moreover, the COVID-19 pandemic caused an increased use of eHealth technologies; thus, in 2021 and 2022, there was an increase in related publications. This work aimed to reflect advances in the matter.

For the conduction of this scoping review, efforts were made to incorporate databases of a complementary nature, which focused, not only on healthcare, but also on exercise and sports. We believe this to have been a relevant decision as efforts were made to provide a general overview of all the existing literature. Having chosen not to restrict the search to any specific language may have helped to strengthen the results, as well.

This scoping review was carried out with a focus on detecting all potential conceptualizations of the term adherence to PA/exercise interventions delivered via eHealth. It contributes, therefore, to narrowing the focus of the concept of adherence to eHealth, and to finding valid, reliable, and acceptable measures of adherence to different physical activity or exercise interventions.

## 7. Conclusions

This review highlightes the fact that a well-defined and unanimous use of the term adherence to exercise is lacking. Traditional approaches which consider adherence as a fraction of prescribed dosage could possibly be suitable for prescription exercise, when performed onsite. Yet, if scientists aim to adhere to the specifications put forth by the WHO, special attention will need to be placed on adequate monitorization of frequency, duration, intensity, and accuracy of the exercises or exercise sessions.

One of the challenges we observed lay in establishing what exactly constituted adherence to voluntary exercise and how it could be measured. Our findings, as well as those by researchers before us, suggest a wide heterogeneity of conceptualizations in the literature. The second challenge we identified had to do with the addition of technologies to interventions, and their remote, asynchronous delivery. Sieverink et al. suggested that “adherence to eHealth technology is an underdeveloped and often improperly used concept”. Our findings, six years later, did not contradict this.

This review calls for an improved definition of adherence to prescribed exercise, adherence to voluntary exercise and adherence to eHealth technologies. This is likely to be a necessary step towards reaching full comparability and applicability.

## Figures and Tables

**Table 1:** Scoping review matrix used for the collection of publication data from the reviewed articles.

Author [Ref]	Abridged Title	Year	Country	Research Design	Technology Type	Outcome	Operationalization
Albergoni [35]	Factors influencing walking and exercise ADH in healthy older adults …	2020	The Netherlands	Exploratory (pilot) study with no control group	The app did not deliver exercises. Served as registry and motivation	Adherence to walking and exercise (difference based on heart rate count) program based on guidelines and adherence to exercise volume	Exercise and walking program ADH *: days per week the target was reached divided by the target training frequency. Exercise volume ADH: minutes per week divided by weekly target volume
Alley [31]	Web-based video-coaching to assist an automated computer-tailored physical activity …	2016	Australia	RCT *	Web-based	ADH to PA intervention and engagement to website	ADH to PA intervention: % of actual vs. planned participation. Website engagement: Number of visits and minutes spent on website
Calvo Sánchez [33]	Experience in the use of videos for the promotion of physical exercise at home in online mode …	2022	Spain	Observational, descriptive, nonexperimental, cross-sectional study	Videos on Vimeo and Youtube platforms	ADH to exercises in videos	Frequency (number per week) of video use; total use time (days)
Dawson [38]	Quantification of adherence in a mobile health exercise intervention for cardiometabolic health	2021	USA	Congress abstract on a pilot trial	Synchronous and asynchronous video sessions online	Adherence to exercise	Exercise ADH: ratio of completed to planned sessions performed/wk. ADH to exercise training dose: through heart rate-based training zones
Feng [37]	Feasibility of an at-home, web-based, interactive exercise program for older adults.	2019	USA	RCT, two-site, single-blind	Secure website	Adherence to exercise intervention	Number of completed sessions divided by total possible sessions (only for finishers).
Jansons [30]	Delivery of home-based exercise interventions in older adults facilitated by Amazon Alexa…	2022	Australia	Feasibility study: prospective single-arm	Amazon Alexa skill application called Teletrainer and Buddy link web-based exercise prescription portal	Adherence to exercise program	Percentage of the total prescribed exercises completed
Jossa-Bastidas [34]	Predicting physical exercise adherence in fitness apps using a deep learning approach	2021	Spain	Observational, retrospective, pilot study	Mobile app	Prediction model	Original app did not define ADH. This retrospective study considered participants as nonadherent if training was interrupted for 4+ continuous weeks
Konstantinidis [32]	Design, implementation, and wide pilot deployment of FitForAll…	2016	Greece	RCT	Computer based with Nintendo Wii devices	Adherence to exercise protocol	Number of participation sessions over the number of planned sessions (%)
Liang [39]	Feasibility and acceptability of home-based exercise snacking and tai chi snacking …	2022	United Kingdom	RCT	Written and video instructions delivered via email	Adherence to exercise program	Percentage of completed vs. prescribed intervention exercises
Morris [28]	Rise and recharge: exploring employee perceptions of and contextual factors …	2021	United Kingdom	Feasibility study: 3-arm quasi-randomized intervention	Mobile app	Adherence to app-delivered prompts	Operationalization of ADH not specified. Study mentioned operationalization for participant compliance to both prompt and review questions, as calculated as the total number of participant responses (reported).
NCT02844296 [40]	Short-bout handgrip exercise for smoking cessation	2016	China	RCT protocol: single-blinded, two-arm	Mobile app with a single instructional video, used to send reminders and as an electronic daily diary	Adherence to suggested single exercise (PA is additionally assessed but not part of the intervention)	Frequency and duration of a single exercise (handgrip).
Pischke [36]	Implementation and effects of information technology-based and print-based interventions …	2020	Germany	RCT protocol with crossover design	Study compared program internet vs. printed-out delivery modes, both modes being complemented with in-person sessions	Changes in PA levels	Adherence measures or operationalization not mentioned. Study used WHO guidelines as a reference
Sun [41]	Motivating adherence to exercise plans through a personalized mobile health app…	2021	Taiwan	Formative research	Mobile app	Adherence to exercise plan	Percentage of the exercise plan completed

* RCT: Randomized controlled trial. * ADH: Adherence.

**Table 2:** Operationalization of reviewed papers as per Yang et al. and Sieverink et al.’s classifications of adherence to eHealth technologies.

Author	Dimension as per Yang et al. [8]	Categorization as per Sieverink et al. [7]
Albergoni [35]	N/a *: App did not deliver exercises. App registered minutes of exercise as recorded by wearable device. Adherence measured in minutes.	N/a. App did not deliver intervention.
Alley [31]	N/a: Internet, not app.	Category A (for web use): Expected use not specified/the more use the better.
Calvo Sánchez [33]	N/a: Video platform, not app.	Category A (for video platform): expected use not specified.
Dawson [38]	N/a: Video platform, not app.	Category A (for videos): expected use not specified/the more use the better.
Feng [37]	N/a: Internet, not app.	Category B (for web use): Expected use specified, not justified.
Jansons [30]	N/a: Amazon Alexa, not app.	Category C (for Alexa plus video program): expected number of exercises specified, as justified by exercise recommendations.
Jossa-Bastidas [34]	N/a. Retrospective study.	N/a. Retrospective study.
Konstantinidis [32]	N/a: Computer, not app.	Category A (for computer use): Expected use of technology not specified.
Liang [39]	N/a: video platform, not app.	Category A (for exercises and prompting): expected use not specified/the more use the better.
NCT02844296 [40]	N/a: Study app was used for participant feedback, not intervention.	N/a. App does not deliver intervention. For participant motivation and feedback purposes: Cat. B—Expected use of “registry” app specified but not justified.
Morris [28]	N/a. Adherence to app not assessed.	Category A (for app use): expected use not specified.
Pischke [36]	N/a: Internet, not app.	Category A (for web use): Expected use of technology not specified.
Sun [41]	N/a. Adherence to app not assessed.	Category A (for app use): expected technology use not specified.

* N/a: not applicable.

## Data Availability

Raw data of this article are available upon request to corresponding author.

## Abbreviations

ADH	adherence
eHealth	electronic health
DHI	Digital health intervention
MeSH	medical subject heading
PA	physical activity
RCT	randomized controlled trial
WHO	World Health Organization

## Appendix A

Keyword terms used within scientific database searches to determine the definitions of adherence to physical activity or physical exercise interventions delivered via eHealth.

**Technology**	**Intervention**	**Adherence**
“Mobile Health”	Exercis*	*Adheren*
mHealth	Train*	“treatment adherence and compliance”
“Mobile tech*”	Program*	
TeleHealth	Coach*	
eHealth	“Physical activity”	
Web*	“Physical fitness”	
Online*	“Resistance training”	
“information tech*”	“High-intensity interval training”	
Wearable*		
smartwatch		
Platform*		
“internet*based”		
App*		

Following are the exact search queries that were used for the retrieval of information in the different databases:

*Cochrane Library:*

(Exercis* OR “Physical activity” OR “Physical fitness” OR “Resistance training” OR “High-Intensity Interval Training”) AND (*Adheren*) AND (teleHealth OR eHealth OR wearable* OR smartwatch OR APP* OR mHealth OR web)

Applied filters: Title Abstract Keywords; 1 January 2016 to 13 March 2022

*Science Direct:*

Adherence AND (Exercise OR “Physical activity”) AND (APP OR eHealth OR mHealth OR Web OR online)

Applied filter: Title Abstract Keywords

*Scielo:*

(Exercis* OR “Physical activity” OR “Physical fitness” OR “Resistance training” OR “High-Intensity Interval Training”) AND (*Adheren*) AND (teleHealth OR eHealth OR wearable* OR smartwatch OR APP* OR mHealth OR web)

Applied filters: none

*Sport Discus:*

(Exercis* OR “Physical activity” OR “Physical fitness” OR “Resistance training” OR “High-Intensity Interval Training”) AND (*Adheren*) AND (teleHealth OR eHealth OR wearable* OR smartwatch OR APP* OR mHealth OR web)

Filter applied: 2016 to 2022

*PubMed:*

(Exercis* OR “Physical activity” OR “Physical fitness” OR “Resistance training” OR “High-Intensity Interval Training”) AND (*Adheren*) AND (teleHealth OR eHealth OR wearable* OR smartwatch OR APP* OR mHealth OR web)

Filters applied: Humans; 1 January 2016 to 13 March 2022; 13 to 80+ years of age; all paper types including reviews.

*Opengrey/Greynet International:*

All attempted search queries retrieved zero relevant literature.
